# TRANSLATING AGING: REACHING STUDENTS ACROSS DISCIPLINES AND GEOGRAPHY

**DOI:** 10.1093/geroni/igad104.1213

**Published:** 2023-12-21

**Authors:** Lisa Borrero

**Affiliations:** University of Indianapolis, Indianapolis, Indiana, United States

## Abstract

What does it mean to translate gerontological concepts among groups of very different students? How can this be optimized for online graduate education? Even within a field that is itself interdisciplinary, it can feel challenging to meaningfully convey key information about aging when students are already firmly established in their own, unique disciplines and are learning from a distance. This lecture will reflect on ways in which gerontology instructors can connect a wide array of graduate students to the universality of aging, promote the relevance of aging to their chosen field, and, hopefully, engender a deeper interest in working with older adults. From the importance of maintaining social and cognitive presence in the online classroom to offering experiential engagement with course content, we can communicate about aging in purposeful and novel ways to a variety of students – and reap the benefits of learning from their expertise in return.

